# Ionic Liquid Composite Polybenzimidazol Membranes for High Temperature PEMFC Applications

**DOI:** 10.3390/polym11040732

**Published:** 2019-04-22

**Authors:** Jorge Escorihuela, Abel García-Bernabé, Álvaro Montero, Óscar Sahuquillo, Enrique Giménez, Vicente Compañ

**Affiliations:** 1 Departamento de Termodinámica Aplicada, (ETSII) Universitat Politècnica de València, Camino de Vera s/n, 46022 Valencia, Spain; agarciab@ter.upv.es (A.G.-B.); almonter@upvnet.upv.es (Á.M.); 2 Departament de Química Orgànica, Universitat de València, Av. Vicent Andrés Estellés s/n, 46100 Burjassot, Valencia, Spain; 3 Instituto de Tecnología de Materiales, Universitat Politècnica de València, Camino de Vera s/n, 46022 Valencia, Spain; ossana@upvnet.upv.es (Ó.S.); enrique.gimenez@mcm.upv.es (E.G.)

**Keywords:** fuel cells, materials science, proton exchange membrane, polymer, polybenzimidazole, ionic liquid, proton conductivity, electrochemical impedance spectroscopy

## Abstract

A series of proton exchange membranes based on polybenzimidazole (PBI) were prepared using the low cost ionic liquids (ILs) derived from 1-butyl-3-methylimidazolium (BMIM) bearing different anions as conductive fillers in the polymeric matrix with the aim of enhancing the proton conductivity of PBI membranes. The composite membranes prepared by casting method (containing 5 wt. % of IL) exhibited good thermal, dimensional, mechanical, and oxidative stability for fuel cell applications. The effects of anion, temperature on the proton conductivity of phosphoric acid-doped membranes were systematically investigated by electrochemical impedance spectroscopy. The PBI composite membranes containing 1-butyl-3-methylimidazolium-derived ionic liquids exhibited high proton conductivity of 0.098 S·cm^−1^ at 120 °C when tetrafluoroborate anion was present in the polymeric matrix. This conductivity enhancement might be attributed to the formed hydrogen-bond networks between the IL molecules and the phosphoric acid molecules distributed along the polymeric matrix.

## 1. Introduction

In the last decades, the scientific community is more concerned about the environmental impact caused by the use of fossil fuels as an energy source. In this scenario, fuel cells have emerged as a new kind of energy transformation device and have been considered as a sustainable and environmentally friendly energy conversion procedure [1]. Among the different variety of fuel cell types, proton exchange membrane fuel cells (commonly referred to as polymer electrolyte membrane fuel cells, PEMFCs) are electrochemical devices that convert chemical energy from a fuel and oxygen into electrical energy, and they have recently attracted increasing interest from fundamental and an applied science due to their future potential as clean and portable power sources [2,3,4,5]. In a typical PEMFC, the polymer electrolyte membrane (PEM) constitutes the fundamental part of the fuel cell technology as it is responsible for the necessary ions conduction between the anode and cathode [6]. Among the different PEMs, perfluorosulfonate acid (PFSA) membranes have received much attention along the last decades because they provide high power density at operating temperatures below 80 °C [7], and particularly Nafion has been the most widely used ionomer for electrochemical applications [8]. Although Nafion currently dominates the fuel cell market, reaching proton conductivities in the range of 0.1 S·cm^−1^ [9], the serious drawbacks, such as temperature effects on the mechanical properties [10] and the proton conductivity decrease at temperatures over 80 °C, have driven efforts for the quest for new polymer electrolytes, which are capable of operating at high temperatures (>100 °C) with high conductivity values under low or anhydrous conditions [11,12].

Energy applications in general, and in particular the automotive industry, need effective polymer electrolyte membranes capable of working at higher temperatures (in the window of 120–140 °C); PEMFCs working within this temperature range are called high temperature proton exchange membrane fuel cells (HT–PEMFCs) [13,14,15,16]. HT–PEMFCs offer several advantages over PEMFCs working at lower temperatures, such as simple heat and water management and high tolerance of CO in the fuel [17]. In this context, it is known that a fuel cell operating pressure of 1.5 bar leads to the requirement of fuel cell components that can operate with 25% relative humidity (RH) at 120 °C because only a 0.5 bar water vapor partial pressure is tolerated [18]. In the quest for developing low cost PEMs with high conductivity at moderate and elevated temperatures, the use of non-perfluorinated polymers has emerged as an alternative to Nafion membranes [19]. In this regard, the main difficulty is to develop PEMs with high proton conductivity, high chemical stability, and high mechanical strength at high temperatures. Among all non-perfluorinated membranes, polybenzimidazole (PBI) has emerged as an attractive candidate to replace Nafion membranes due to its superior thermal and mechanical stability [20]. PBI membranes exhibit low proton conductivities under low humidity conditions. However, they can be significantly improved when doped with phosphoric acid (PA), showing conductivities up to 0.08 S·cm^−1^ at 150 °C, which is as high as a wetted Nafion membrane [21]. Although phosphoric acid doping enhances proton conductivity, its use has some drawbacks regarding acid leaking and phosphoric acid degradation over 160 °C, resulting in a decrease of proton conductivity. As a consequence, significant efforts have been focused on the search for new approaches to enhance PBI conductivity. In the last years, different approaches were developed in order to overcome this problem and improve the mechanical/dimensional properties and proton conductivity performance of PBI membranes at elevated temperatures by using several fillers such as silica and clay [22,23], metalcarborane and metal oxides [24], phosphate salts [25], heteropolyacids [26], metal organic frameworks (MOFs) [27,28], graphene oxide (GO), [29,30] and more recently, ionic liquids [31,32].

Ionic liquids (ILs) are molten salts composed of organic cations and inorganic anions with low melting points around room temperature (below 100 °C) [33,34]. ILs are promising compounds for the preparation of electrochemical devices because they exhibit high thermal stability, good conductivities and low or even neglected leaching of the bulk IL component has been detected when the ILs is taking part in a polymeric matrix [35,36]. In recent years, the use of ILs as fillers in polymer-based membranes has been deeply studied in gas separation processes [37], drug sensing [38], pharmaceutics and medicine [39], transport agents [40], catalysis [41,42,43], and energy storage and conversion [44,45,46]. ILs offer a significant advantage over phosphoric acid doping as they are highly stable at temperatures higher than 160 °C. One of the biggest advantages of ILs is their potential application as a filler of novel polymeric membranes that combines the good properties of the ILs with those of polymers to build composite polyelectrolytes as polymer ionic liquids (PILs) [47].

Herein, in this work we have prepared different proton conducting PBI composite membranes by incorporating 1-butyl-3-methylimidazolium (BMIM)-derived IL bearing different anionic units ([Cl]^−^, [Br]^−^, [I]^−^, [NCS]^−^, [NTf_2_]^−^, [PF_6_]^−^ and [BF_4_]^−^) in the polymeric network. Composite membranes with different anions were prepared by solution casting and the structure, morphology, thermal stability, mechanical strength, oxidative resistance and proton conductivity of these prepared materials were analyzed to study their sustainability to be used as HT–PEMFCs. The incorporation of ionic liquids as fillers in PBI membranes improves the proton conductivity, with values up to 94 mS·cm^−1^ being obtained for the corresponding composite membrane containing BMIM-BF4 at 200 °C under anhydrous conditions.

## 2. Materials and Methods 

### 2.1. Materials.

PBI (purity > 99.95%, MW 51000, with the molecular formula: (C_20_H_12_N_4_)*_n_*) was purchased from Danish Power Systems (Danish Power Systems, Kvistgaard, Denmark). LiCl, *N*,*N*-dimethylacetamide (DMAc) 99.8%, 1-butyl-3-methylimidazolium chloride (BMIM–Cl), 1-butyl-3-methylimidazolium bromide (BMIM–Br), 1-butyl-3-methylimidazolium iodide (BMIM–I), 1-butyl-3-methylimidazolium tetrafluoroborate (BMIM–BF_4_), 1-butyl-3-methylimidazolium hexafluorophosphate (BMIM–PF_6_), 1-butyl-3-methylimidazolium bis(trifluoromethylsulfonyl)imide (BMIM–NTf_2_), and 1-butyl-3-methylimidazolium thiocyanate (BMIM–NCS) were purchased from Sigma–Aldrich (Sigma–Aldrich Química SL, Madrid, Spain). *N*,*N*–Dimethylacetamide (DMAc, 99.5% extra pure) and phosphoric acid (extra pure, 85% solution in water) were purchased from Acros Organics (Fisher Scientific SL, Madrid, Spain). 

### 2.2. Characterization

Scanning electron microscopy (SEM) images were acquired on a field emission scanning electron microscope (FE–SEM) model Ultra 55 (Zeiss, Oberkochen, Germany) operating at 5 kV with energy-dispersive X-ray (EDX) spectroscopy. Electron micrographs were obtained using a Jeol JEM–1010 high resolution microscope (JEOL Ltd., Garden City, UK). Attenuated total reflection Fourier transform infrared (ATR–FTIR) spectra of the membranes were recorded on a Jasco FT–IR spectrometer FT/IR-6200 Series (Jasco Spain, Madrid, Spain) with a 4 cm^−1^ resolution between 400 and 4000 cm^−1^. Thermogravimetric analysis (TGA) was performed on a TGA Q50 thermogravimetric analyzer TGA Q50 (Waters Cromatografia, S.A., Division TA Instruments, Cerdanyola del Valles, Spain). The samples (5–10 mg) were weighed in platinum crucibles and were heated under nitrogen atmosphere (60 mL·min^−1^) from room temperature to 800 °C at a heating rate of 10 °C·min^−1^. For the surface area and porosity analysis, the solid or membrane was dried in a vacuum oven at 100 °C for 5 h and activated at 100 °C for 12 h on a SmartVacPrep instrument (Micromeritics Instrument Corporation, Norcross, GA, USA). The acid uptake (AU) of the membrane was calculated by the following equation: AU (%) = [(*W*_wet_ − *W*_dry_)/*W*_dry_] × 100; where *W*_wet_ and *W*_dry_ refer to the membrane’s weight after its immersion in phosphoric acid for at least 48 h at room temperature and the membrane’s weight after drying at 120 °C for at least 24 h, respectively. The oxidative stability (OS) of the membranes was investigated by immersing the membranes in Fenton’s reagent (3% H_2_O_2_ solution containing 4 ppm Fe^2+^) at 70 °C. The samples were collected by filtering and rinsing with deionized water several times, then dried at 120 °C for 5 h in a vacuum oven. Next, the degradation of the membranes was evaluated by their weight loss by using the following equation: OS (%) = [(*W*_1_ − *W*_2_)/*W*_1_] × 100; where *W*_1_ is the weight of the dried membrane before the Fenton test and *W*_2_ is the weight of the dried membrane after the Fenton test. The tensile properties of the membranes were determined from stress–strain curves obtained with a universal testing machine (Shimadzu AGS-X) at a crosshead rate of 10 mm·min^−1^ at room temperature. The membranes were cut into strips of 30 mm × 6 mm and were around 100 µm thick. Five specimens of each sample were tested and the average results with standard deviation were reported. The proton conductivity measurements of the membranes in the transversal direction were performed in the temperature range between 0 and 200 °C by electrochemical impedance spectroscopy (EIS) in the frequency interval of 0.1 Hz to 10 MHz, applying a 0.1 V signal amplitude. A broadband dielectric spectrometer (Novocontrol Technologies, Hundsangen, Germany) integrated with an SR 830 lock-in amplifier with an Alpha dielectric interface was used. The membranes were previously immersed in deionized water and the thickness was measured afterwards using a digital micrometer, taking the average measurements at different parts of the surface. Then, the membranes were placed between two gold electrodes coupled to the spectrometer. Initially, the temperature was gradually raised from 20 to 120 °C in steps of 10 °C and the dielectric spectra were collected at each step. During the second cycle of temperature scan (named as anhydrous conditions), the dielectric spectra were collected at each step. In addition, during the second cycle of the temperature scan (called as dry conditions in the manuscript), the dielectric spectra were collected at each step from 0 to 200 °C, in steps of 10 °C.

### 2.3. Experimental Procedures

#### 2.3.1. Preparation of the PBI Solution

LiCl (0.1 wt. %) was used as a stabilizer and was dissolved in DMAc with vigorous stirring (1 h at room temperature) to give a homogeneous solution. Next, PBI powder (10 wt. %) was dissolved in the LiCl solution (in DMAc) and heated under reflux at 120 °C for 6 h. The prepared solution had a viscosity of 0.5 Pa·s at 25 °C.

#### 2.3.2. Membrane Preparation

The amount of 0.025 g of ionic liquid was totally dissolved in 4.975 g of the 10 wt. % PBI solution and placed in an ultrasonic bath for 20 min to give a final solution with a 5 wt. % of IL with respect to PBI. Next, the homogeneous solution was stirred for 4 h at 60 °C. Then, the solution was cast onto a glass slide and dried at 70 °C for 10 h, then at 140 °C for 10 h, and finally at 120 °C under vacuum overnight. Membranes were washed with distilled water at 80 °C in order to remove residual solvent (DMAc). Traces of the solvent were finally removed by drying at 160 °C for 16 h. The membrane thicknesses prior to acid doping varied between 190 and 210 μm.

## 3. Results and discussion

**Figure 1:**
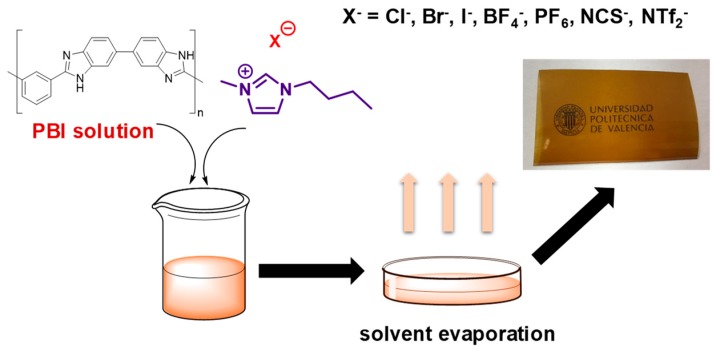
Schematic representation of PBI composite membranes containing ionic liquids and photograph of PBI@BMIM-NTf_2_ membrane.

Composite PBI membranes containing ILs were prepared by casting method (Figure 1). For this purpose, the amount of ionic liquid (0.05 g) was dissolved in the 10 wt. % PBI solution (10 g) under vigorous stirring to give the PBI solution containing 5 wt. % of ionic liquid. This solution was cast onto a glass plate and dried at 80 °C for 8 h; then is was dried at 160 °C for 10 h. Then, membranes were peeled of the glass plate and finally dried under pressure at 140 °C for 10 min. Membranes were washed with distilled water at 80 °C in order to remove residual solvent (DMAc). Traces of the solvent were removed by drying at 160 °C for 16 h. The membrane thicknesses prior to acid doping varied between 190 and 210 μm.

**Figure 2:**
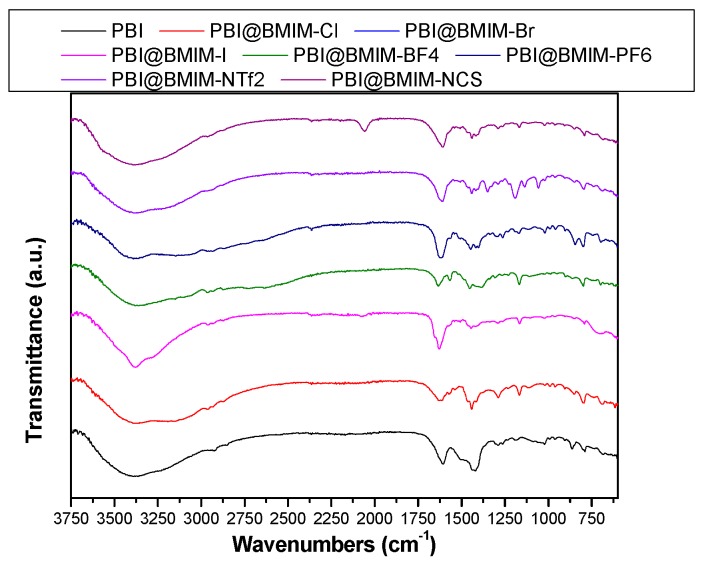
FT–IR spectra of PBI and PBI composite membranes containing different ionic liquids derived from BMIM (5 wt. %).

Infrared spectroscopy is highly useful for studies on materials science. Figure 2 shows the FT–IR spectra of the 5 wt. % IL–PBI composite membranes in the range of 4000–600 cm^−1^. The pure PBI membrane showed a typical broad peak around 3500–3200 cm^−1^ attributed to the N–H stretching, and two bands at 1610 and 1423 cm^−1^, which are associated with C=N and C–N stretching vibrations, respectively [48]. After incorporation of the IL in the polymer matrix, the presence of 1-butyl-3-methylimidazolium bis(trifluoromethylsulfonyl)imide in the membranes was confirmed by the presence of peaks at 1192 cm^−1^ (CF_3_ stretching), 1591 cm^−1^ (SO_2_ asymmetric stretching), 1131 cm^−1^ (SO_2_ symmetric stretching), and 1052 cm^−1^ (S–N stretching) [49]. For the PBI membrane containing 5 wt. % BMIM-NCS, a characteristic band from the thiocyanate group was observed at 2058 cm^−1^. A shift of IR peak at 1608 cm^−1^ to a higher wavenumber was observed for some ILs under study, attributed to the presence of hydrogen bond interactions between IL and the polymeric matrix [50].

**Figure 3:**
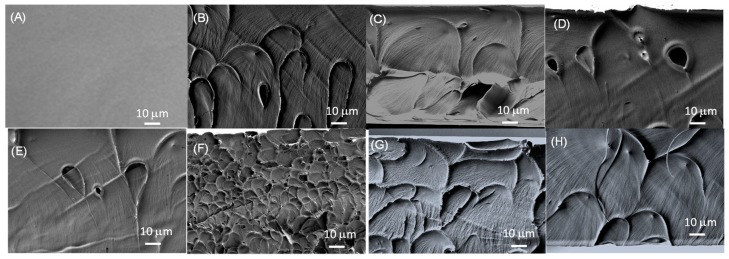
Cross-sectional SEM images of (**A**) PBI, (**B**) PBI@BMIM-Cl, (**C**) PBI@BMIM-Br, (**D**) PBI@BMIM-I, (**E**) PBI@BMIM-NCS, (**F**) PBI@BMIM-BF_4_, (**G**) PBI@BMIM-PF_6_, and (**H**) PBI@BMIM-NTf_2_.

The internal microscopic morphologies of membranes were studied by SEM. The cryofractured cross sections of the different PBI composite membranes containing 5 wt. % of ionic liquid are shown in Figure 3. The surface of the PBI membranes was dense and free of holes. However, the addition of ILs as fillers was reflected in the appearance of holes in the cross section SEM images. After PA doping, the morphology of all membranes showed the formation of channels due to the presence of PA in the polymer network, as observed in similar systems [51]. After immersion in H_3_PO_4_ aqueous solution (15 M), acid uptake (AU) was calculated from weight difference and values around 243%–256% were obtained for composite membranes containing 5 wt. % of ionic liquid. It is worth mentioning that the prepared polymeric membranes are stable and no coloration was observed even in 15 M H_3_PO_4_.

**Figure 4:**
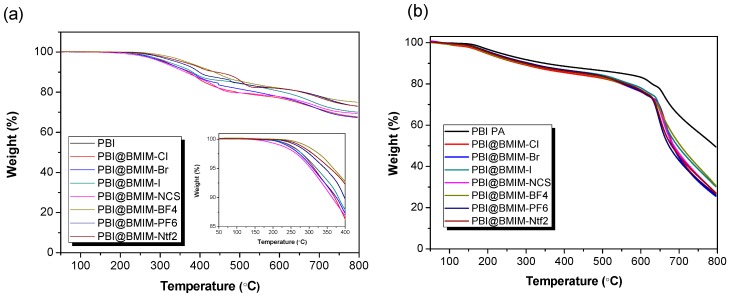
TGA curves of (**a**) undoped and (**b**) phosphoric acid-doped PBI composite membranes containing different ionic liquids derived from BMIM (5 wt. %) under a N_2_ atmosphere.

High stability at elevated temperatures is one of the requirements that ideal polymer electrolyte membranes must fulfil in order to guarantee proton transport. The thermal properties of undoped PBI-based membranes containing ionic liquids derived from BMIM (5 wt. %) with different anions were studied by TGA under a N_2_ atmosphere (Figure 4a). For the pure PBI membrane, about 5% loss occurred in the range of temperature from 50 to 250 °C, which is attributed to the dehydration of absorbed water molecules and traces of DMAc. Finally, polymeric backbone degradation occurs around 710 °C. All PBI composite membranes containing 5 wt. % of ionic liquid showed high thermal stability up to 200 °C, with a with a weight loss of 3%–8% depending on the anion of BMIM. In the 250–500 °C range of decomposition temperatures also occurs the thermal degradation of ionic liquids incorporated into the PBI membranes, as decomposition of BMIM anion occurred in the range 350–500 °C [52]. This decomposition is partially masked with primary polymer degradation. The degradation step observed at about 600 °C was associated with the degradation of the PBI main chain [53]. After several decomposition stages, composite membranes remained with 70%–76% weight at 800 °C, slightly lower than pure PBI membrane (79% weight). Comparing with the undoped membranes, the weight loss curves for the PA-doped membranes showed a similar degradation trend (Figure 4b). The first weight loss step was observed at 160–165 °C due to the PA dehydration and the subsequent formation of pyrophosphoric acid (H_4_P_2_O_7_) [54,55] and more complex phosphate species at higher temperature, as previously reported in similar PA-doped PBI membranes [56,57,58]. The T_d,95_ (where the weight loss reaches 5 wt. %) of PBI composite membranes was around 300–350 °C. After PA doping, a thermal stability reduction was observed compared to undoped membranes, as shown by the *T*_d,95_, which was lowered to 180–210 °C for doped membranes, which results from the PA dehydration and formation of pyrophosphoric acid or other phosphate species. From the results, it can be concluded that the composite membranes reported in this study possess enough thermal stability for its application as high temperature proton exchange membrane fuel cells.

**Figure 5:**
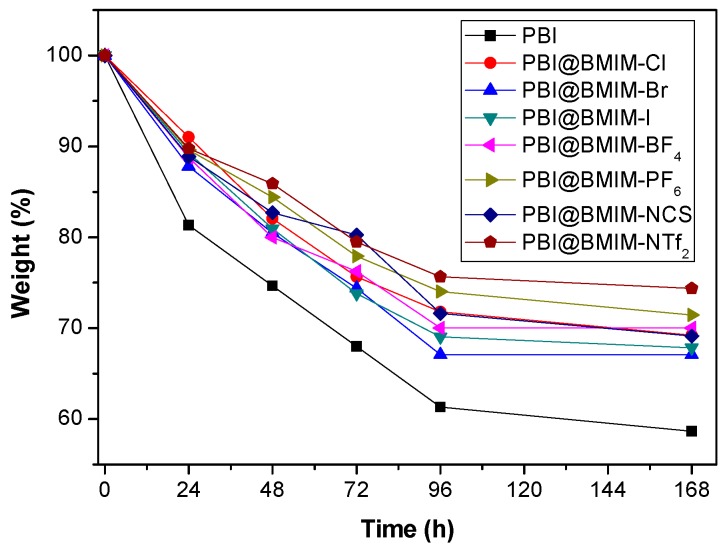
Weight loss of the IL composite membranes (containing 5 wt. % of BMIM-X) and PBI after Fenton test.

During the operation of PEMFCs, the polymeric membrane can be degraded by radicals; therefore, the evaluation of stability is a necessary test when developing novel PEMs. In this work, the oxidative stability of the composite membranes containing 5 wt. % of IL was evaluated by weight loss in relation to the initial weight after immersion in the Fenton’s solution [59]. The pure PBI membrane showed a weight decrease around 20% after 24 h and 40% after 196 h (1 week). In contrast, the weight decrease of all composite membranes was below 15 and 30% after 24 and 196 h, respectively (Figure 5). The addition of the IL enables a crosslinking network, which improves the stability of the composite membranes as already observed for other blend membranes. The oxidative stabilities of composite membranes are in the same range as those reported for other non-fluorinated analogs in literature [60].

The mechanical properties of the undoped PBI composite membranes containing 5 wt. % of BMIM-X were evaluated by tensile testing. A summary of the tensile testing results is shown in Table 1. In all cases, the Young’s modulus and tensile strength of the composite membranes increased with the addition of the ionic liquid (5 wt. %) as filler, indicating that the ILs can improve the mechanical properties of PBI by an interaction between the polymer matrix and the absorbed ILs. It should be noted that the composite with BMIM-Cl showed a significant increase in the values of Young’s modulus and tensile strength with respect to pure PBI (2.6 GPa and 97 MPa, respectively), up to 2.8–3.7 GPa and 124–141 MPa, respectively. However, the strain at break decreases with IL loading, indicating a higher brittle in the composite membranes than in the PBI alone. Moreover, the membrane PBI-BMIM-NTF_2_ showed a good balance of mechanical properties, by offering high strength and a lower decrease in strain at break compared with the other composite membranes studied [61]. After the acid doping, the stress–strain curves for these composite materials exhibited a rubbery nature (a decrease in tensile strength and Young’s modulus but an increase in the strain at break was observed), due to the uptake of H_3_PO_4_; contrarily to shows that exhibited a glassy nature all the undoped membranes [62].

**Figure 6:**
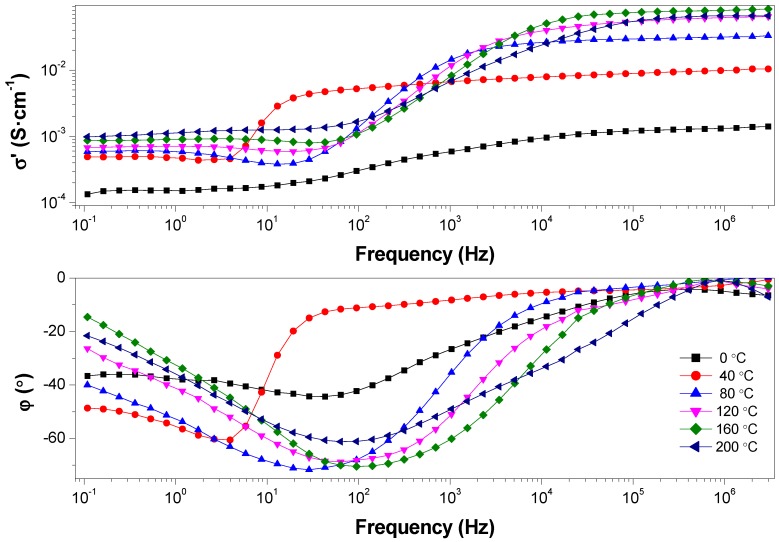
Bode diagram for phosphoric acid-doped PBI@BMIM-NTf_2_ composite membrane (containing 5 wt. % of BMIM-NTf_2_) under anhydrous conditions. In the top graphical representation, σ′ is plotted against the frequency, whereas in the bottom, the out of phase angle φ is plotted against the frequency.

Proton conductivity of composite membranes was evaluated by electrochemical impedance spectroscopy (EIS). In the last decades, this electrochemical technique has been applied to measure the through-plane conductivity of PEMs [63,64]. The proton transport was determined by investigating the dependence of proton conductivity on the temperature under anhydrous conditions from 0 to 200 °C (see Supplementary Information, Table S1). The dc-conductivity for the composite membranes was obtained by means of the Bode diagram [65,66]. In this graphical representation for PBI@BMIM-NTf_2_ (see Figure 6), the modulus of the complex impedance |σ*| is plotted against the frequency ω (i.e., |Z*| vs. ω). In a typical Bode diagram, as frequency increases, the modulus increases, reaching a plateau at a given frequency (σ′ is constant with the frequency), whereas the out of phase angle φ = tan^−1^ (Z″/Z′) reaches a maximum (or generally tends to zero). Since lim |Z*| → R_0_ and φ = 0 at ω → ∞, the ionic resistance is R_0_ = |Z*| at tan^−1^ (Z″/Z′) = 0, and then the dc-conductivity is the constant value obtained from the plateau. The dc-conductivity (σ_dc_, S·cm^−1^) is related with the impedance of the membrane by means of (σ_dc_ = L/(R_0_·S), where L (cm) is the thickness of the membrane, A (cm^2^) is the contact surface area between the electrodes and the membrane, and R_0_ (Ω) is the membrane resistance.

As can be seen in Figure 6, the through-plane conductivities increased with temperature for the composite membranes, showing a linear temperature dependence between 0 and 100 °C. In some cases, a decrease in conductivity was observed for temperatures over 160 °C due to the evaporation of phosphoric acid. As shown, proton conductivity showed a strong dependence on the anion present in the IL of the composite membrane. As an example (see Table 2), BMIM membranes containing halogen anions (Cl, Br or I) displayed lower conductivity values than pristine PBI membrane at temperatures below 80 °C, with conductivities at 200 °C of 26, 58 and 7 mS·cm^−1^, for PBI@BMIM-Cl, PBI@BMIM-Br and PBI@BMIM-I, respectively. Composite membranes PBI@BMIM-PF_6_ and PBI@BMIM-NCS showed similar conductivities, reaching maximum values of 23 and 26 mS·cm^−1^ at 200 °C, respectively. When comparing composite membranes PBI@BMIM-BF_4_ and PBI@BMIM-PF_6_, the dc-conductivity for the former membrane was around four-fold higher over the whole temperature range, which might be attributed to the higher hydrophobicity of ionic liquid containing [PF_6_]^−^ anion, as hydrophobicity is dependent on the number of F atoms. Membranes containing anions such as [NTf_2_]^−^ and [BF_4_]^−^ displayed the highest conductivities with values at 200 °C of 65 and 94 mS·cm^−1^, for PBI@BMIM-NTf_2_ and PBI@BMIM-BF_4_, respectively. The influence of the anion on ionic liquid over the conductivity of the composite membranes can be interpreted in terms of the changes in polarity and hygroscopicity associated to the anion [67]. These values are similar to other reported PBI composite membranes containing ionic liquids under anhydrous conditions [68]. Although the experimental procedure was designed in order to eliminate the adsorbed water on the composite membranes by performing a previous cycle from 20 to 120 °C before each measurement, this effect cannot be completely ruled out, as traces of water molecules can be retained in the polymeric matrix due to strong associations with the ionic liquids incorporated in the polymer.

**Figure 7:**
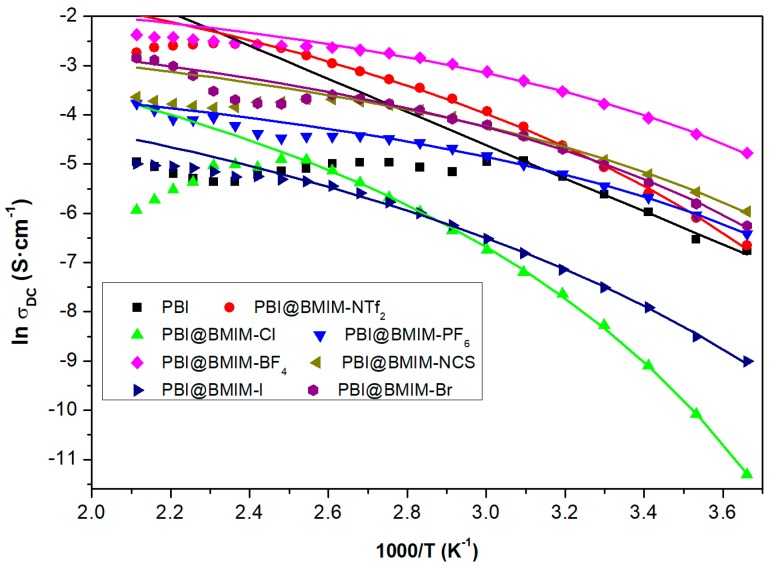
Representation of the ln of conductivity (σ_dc_) as a function of the reciprocal of the temperature for phosphoric acid-doped PBI composite membranes containing 5 wt. % of BMIM-X.

A closer inspection of the variation of the values of conductivity with the temperature according to an Arrhenius plot (ln σ vs 1000/T) is shown in Figure 7. In order to further study the proton conduction mechanism of the PA-doped composite membranes, the activation energy (E_act_) was calculated. From this plot, it is evident that the activation energy associated to the conductivity mechanism is not constant over the whole range of temperatures. The activation energy is much higher at lower temperatures than at the higher. In agreement with our experimental results, we have obtained the temperature dependence of the conductivity according to a Vogel–Fulcher–Tammann (VFT) equation given by:

(1) $$\log\sigma =\log\sigma_{\infty }-\frac{E_{\mathrm{act}}}{R\left(T-T_{0}\right)}$$

where σ is the proton conductivity in S cm^−1^, σ_0_ is the preexponential factor, E_act_ is the activation energy of the process underlying the dc-conductivity (σ_dc_), and *R* is the gas constant (8.314 J·mol^−1^·K^−1^). Notice that E_act_/*R* is a fitting parameter related with the curvature of the plot identical to the VFT parameter with units of temperature in Kelvin, and *T*_0_ is the Vogel temperature, considered as the one at which the relaxation time would diverge, and σ_∞_ is a pre-factor related with the limit conductivity at higher temperatures. 

The corresponding values obtained for the VFT parameters, T_0_ and σ_∞_, are shown in Table 3. In order to study in detail the proton conduction mechanism of the PA-doped composite membranes, the activation energy (E_act_) was calculated. The calculated values for the activation energy for IL-containing PBI membranes decrease according to the following trend [Cl]^−^ > [I]^−^ > [NTf_2_]^−^ > [Br]^−^ > [NCS]^−^ > [BF_4_]^−^ ≈ [PF_6_]^−^, and were in the range of 2.5–6.3 kJ·mol^−1^, which are lower compared to other reported values of PA-doped PBI membranes [69,70,71] and lower for that obtained for the pristine PBI membrane (26.8 kJ·mol^−1^).

As seen from the Arrhenius plot in Figure 7, the addition of 5% BMIN-Cl and BMIN-I to the PBI matrix shows a decrease of conductivity in comparison with the pristine PBI [71]. However, the incorporation of the other ILs produces an important increase of conductivity when the membrane is doped with 15 M phosphoric acid. This variation may be related with the coulomb energy of the cation–anion pair present in the ionic liquid, which is determined by the temperature dependence of the free ion concentration in the polymeric matrix. It is known that the conductivity of a polymer electrolyte can be described by the Einstein expression as σ = nqμ, where n is the free charge density, q is the charge of a monovalent ion, and μ its mobility [72]. Considering that n is temperature dependent, n(T), and knowing that the mobility of free ions is expected to be controlled by the segmental motion of the polymeric matrix of PBI, which in turn will depend on the temperature, μ(T). The real temperature dependence of conductivity will be under the influence of both dependences. Consequently, the expression shown in Equation (1) will be only an approximation to the real prediction of temperature dependency of the conductivity. From the fits, we find ionic conductivity to be in reasonable agreement with Equation (1), resulting in that the curvature of the fit in conductivity originates from VFT temperature dependence could be more strongly associate to the ionic mobility than charge density. From our results, we can see that at 120 °C, the conductivity varies between 4.7 × 10^−4^ and 6.2 × 10^−2^ S·cm^−1^ depending on the type of anion. These values are goods as a polymer electrolyte to be applied in fuel cells to work at moderate and high temperatures, at least in the range of 120–200 °C.

## 4. Conclusions

In summary, this contribution presents a series of proton exchange membranes based on polybenzimidazole (PBI) enhanced using the low cost ionic liquids (ILs) derived from 1-butyl-3-methylimidazolium (BMIM) as conductive fillers in the polymeric matrix. The incorporation of ionic liquids as fillers in PBI membranes improves the mechanical properties of the composite membrane by an interaction between the polymer matrix and the IL. In this regard, conductivities up to 94 mS·cm^−1^ have been obtained for the corresponding composite membrane containing BMIM-BF4 at 200 °C under anhydrous conditions. These results here presented show that a fine-tuning of polymer composite membranes can be achieved by the proper selection of the ionic liquid used in their preparation. This modular behavior facilitates the optimization process and opens the way for the future development of high-temperature electrolytes for further applications in different fields, in particular as electrochemical devices in energy-related areas.

## Figures and Tables

**Table 1:** Mechanical properties of undoped IL composite PBI membranes (containing 5 wt. % of BMIM-X) studied in this work.

Membrane	Young’s Modulus (GPa)	Tensile Stress (MPa)	Strain at break (%)
PBI	2.6 ± 0.5	97 ± 4	27 ± 4
PBI@BMIM-Cl	3.7 ± 0.1	141 ± 3	9 ± 1
PBI@BMIM-Br	3.0 ± 0.3	128 ± 4	15 ± 3
PBI@BMIM-I	3.6 ± 0.1	131 ± 3	7 ± 1
PBI@BMIM-BF4	2.8 ± 0.1	125 ± 4	17 ± 4
PBI@BMIM-PF6	3.4 ± 0.3	124 ± 2	8 ± 1
PBI@BMIM-NCS	3.6 ± 0.4	131 ± 3	10 ± 1
PBI@BMIM-NTf_2_	3.1 ± 0.3	127 ± 2	19 ± 1

**Table 2:** Conductivity values (in S·cm^−1^) for some temperatures obtained from the Bode diagram for all phosphoric acid-doped PBI composite membranes containing 5 wt. % of BMIM-X under anhydrous conditions.

T (°C)	PBI	[Cl]^−^	[Br]^−^	[I]^−^	[BF_4_]^−^	[PF_6_]^−^	[NCS]^−^	[NTf_2_]^−^
0	1.2 × 10^−3^	1.2 × 10^−5^	1.9 × 10^−3^	1.2 × 10^−4^	8.5 × 10^−3^	1.6 × 10^−3^	2.6 × 10^−3^	1.3 × 10^−3^
40	5.2 × 10^−3^	4.8 × 10^−4^	9.1 × 10^−3^	7.9 × 10^−4^	2.9 × 10^−2^	5.4 × 10^−3^	9.5 × 10^−3^	9.8 × 10^−3^
80	6.3 × 10^−3^	2.6 × 10^−3^	2.0 × 10^−2^	2.5 × 10^−3^	5.8 × 10^−2^	1.0 × 10^−2^	2.0 × 10^−2^	3.1 × 10^−2^
120	6.1 × 10^−3^	7.4 × 10^−3^	2.5 × 10^−2^	4.7 × 10^−4^	7.4 × 10^−2^	1.2 × 10^−2^	2.5 × 10^−2^	6.1 × 10^−2^
160	4.7 × 10^−3^	6.5 × 10^−3^	3.0 × 10^−2^	5.8 × 10^−3^	8.2 × 10^−2^	1.7 × 10^−2^	2.1 × 10^−2^	7.8 × 10^−2^
200	7.1 × 10^−3^	2.6 × 10^−2^	5.8 × 10^−2^	6.8 × 10^−3^	9.4 × 10^−2^	2.3 × 10^−2^	2.6 × 10^−2^	6.5 × 10^−2^

**Table 3:** VFT fitting parameters for the PBI composite membranes under anhydrous conditions studied in this work.

Membrane	Ln σ_∞_ (S·cm^−1^)	*T*_0_ (K)	E_act_ (kJ·mol^−1^)
PBI@BMIM-Cl	−1.02	199	6.33
PBI@BMIM-Br	−1.61	195	3.04
PBI@BMIM-I	−2.19	172	5.80
PBI@BMIM-BF4	−0.97	194	2.53
PBI@BMIM-PF6	−2.72	192	2.51
PBI@BMIM-NCS	−1.81	190	2.91
PBI@BMIM-NTf_2_	0.24	181	5.35

## Supplementary Materials

The following materials are available online at https://www.mdpi.com/2073-4360/11/4/732/s1, Table S1: Conductivity values obtained from the Bode diagram for all phosphoric acid doped PBI composite membranes containing 5 wt. % of BMIM-X under anhydrous conditions; Figure S1: Bode diagram for phosphoric acid doped PBI@BMIM-Cl composite membrane under anhydrous conditions; Figure S2: Bode diagram for phosphoric acid doped PBI@BMIM-Br composite membrane under anhydrous conditions; Figure S3: Bode diagram for phosphoric acid doped PBI@BMIM-I composite membrane under anhydrous conditions; Figure S4: Bode diagram for phosphoric acid doped PBI@BMIM-BF_4_ composite membrane under anhydrous conditions; Figure S5: Bode diagram for phosphoric acid doped PBI@BMIM-PF_6_ composite membrane under anhydrous conditions; Figure S6: Bode diagram for phosphoric acid doped PBI@BMIM-NCS composite membrane under anhydrous conditions.
