# Supplementary material for: Ionic Liquid Composite Polybenzimidazol Membranes for High Temperature PEMFC Applications
Source: Polymers (Basel). 2019 Apr 22;11(4):732. doi: 10.3390/polym11040732 (PMC6523944; doi:10.3390/polym11040732)
Supplement: Supplementary file 1 [file polymers-11-00732-s001.pdf]

Supporting Information

Ionic liquid composite polybenzimidazol membranes for high temperature PEMFC applications

Jorge Escorihuela [1,2], Abel García-Bernabé [1], Álvaro Montero [1], Óscar Sahuquillo [3], Enrique Giménez [3] and Vicente Compañ [1,*]

[1] Departamento de Termodinámica Aplicada (ETSII) Universitat Politècnica de València, Camino de Vera s/n, 46022 Valencia, Spain; escorihu@uji.es (J.E.); agarciab@ter.upv.es (A.G.-B.); almonter@upvnet.upv.es (A.M.)
[2] Departament de Química Orgànica, Universitat de València, Av. Vicent Andrés Estellés s/n, 46100, Burjassot, Valencia, Spain; escorihu@uji.es (J.E)
[3] Instituto de Tecnología de Materiales, Universitat Politècnica de València, Camino de Vera s/n, 46022 Valencia, Spain; ossana@upvnet.upv.es (O.S.); enrique.gimenez@mcm.upv.es (E.G.)
* Correspondence: vicommo@ter.upv.es; Tel.: +34-96-387-9328

1. Supplementary Tables.

Table S1. Conductivity values obtained from the Bode diagram for all phosphoric acid doped PBI composite membranes containing 5 wt. % of BMIM-X under anhydrous conditions.

T (°C)	PBI	$[Cl]^-$	$[Br]^-$	$[I]^-$	$[BF_4]^-$	$[PF_6]^-$	$[NCS]^-$	$[NTf_2]^-$
0	$1.2 \cdot 10^{-3}$	$1.2 \cdot 10^{-5}$	$1.9 \cdot 10^{-3}$	$1.2 \cdot 10^{-4}$	$8.5 \cdot 10^{-3}$	$1.6 \cdot 10^{-3}$	$2.6 \cdot 10^{-3}$	$1.3 \cdot 10^{-3}$
10	$1.5 \cdot 10^{-3}$	$4.2 \cdot 10^{-5}$	$3.0 \cdot 10^{-3}$	$2.0 \cdot 10^{-4}$	$1.2 \cdot 10^{-2}$	$2.4 \cdot 10^{-3}$	$3.8 \cdot 10^{-3}$	$2.2 \cdot 10^{-3}$
20	$2.5 \cdot 10^{-3}$	$1.1 \cdot 10^{-4}$	$4.6 \cdot 10^{-3}$	$3.7 \cdot 10^{-4}$	$1.7 \cdot 10^{-2}$	$3.4 \cdot 10^{-3}$	$5.4 \cdot 10^{-3}$	$3.7 \cdot 10^{-3}$
30	$3.6 \cdot 10^{-3}$	$2.5 \cdot 10^{-4}$	$6.6 \cdot 10^{-3}$	$5.5 \cdot 10^{-4}$	$2.3 \cdot 10^{-2}$	$4.3 \cdot 10^{-3}$	$7.2 \cdot 10^{-3}$	$6.3 \cdot 10^{-3}$
40	$5.2 \cdot 10^{-3}$	$4.8 \cdot 10^{-4}$	$9.1 \cdot 10^{-3}$	$7.9 \cdot 10^{-4}$	$2.9 \cdot 10^{-2}$	$5.4 \cdot 10^{-3}$	$9.5 \cdot 10^{-3}$	$9.8 \cdot 10^{-3}$
50	$7.2 \cdot 10^{-3}$	$7.5 \cdot 10^{-4}$	$1.2 \cdot 10^{-2}$	$1.1 \cdot 10^{-3}$	$3.6 \cdot 10^{-2}$	$6.7 \cdot 10^{-3}$	$1.2 \cdot 10^{-2}$	$1.4 \cdot 10^{-2}$
60	$7.1 \cdot 10^{-3}$	$1.2 \cdot 10^{-3}$	$1.5 \cdot 10^{-2}$	$1.5 \cdot 10^{-3}$	$4.4 \cdot 10^{-2}$	$7.9 \cdot 10^{-3}$	$1.5 \cdot 10^{-2}$	$2.0 \cdot 10^{-2}$
70	$5.8 \cdot 10^{-3}$	$1.7 \cdot 10^{-3}$	$1.7 \cdot 10^{-2}$	$1.9 \cdot 10^{-3}$	$5.1 \cdot 10^{-2}$	$9.3 \cdot 10^{-3}$	$1.8 \cdot 10^{-2}$	$2.5 \cdot 10^{-2}$
80	$6.3 \cdot 10^{-3}$	$2.6 \cdot 10^{-3}$	$2.0 \cdot 10^{-2}$	$2.5 \cdot 10^{-3}$	$5.8 \cdot 10^{-2}$	$1.0 \cdot 10^{-2}$	$2.0 \cdot 10^{-2}$	$3.1 \cdot 10^{-2}$
90	$6.9 \cdot 10^{-3}$	$3.4 \cdot 10^{-3}$	$2.3 \cdot 10^{-2}$	$3.1 \cdot 10^{-3}$	$6.4 \cdot 10^{-2}$	$1.1 \cdot 10^{-2}$	$2.2 \cdot 10^{-2}$	$3.8 \cdot 10^{-2}$
100	$7.0 \cdot 10^{-3}$	$4.6 \cdot 10^{-3}$	$2.5 \cdot 10^{-2}$	$3.7 \cdot 10^{-3}$	$6.8 \cdot 10^{-2}$	$1.2 \cdot 10^{-2}$	$2.4 \cdot 10^{-2}$	$4.4 \cdot 10^{-2}$
110	$6.8 \cdot 10^{-3}$	$5.8 \cdot 10^{-3}$	$2.7 \cdot 10^{-2}$	$4.3 \cdot 10^{-3}$	$7.2 \cdot 10^{-2}$	$1.2 \cdot 10^{-2}$	$2.5 \cdot 10^{-2}$	$5.2 \cdot 10^{-2}$
120	$6.1 \cdot 10^{-3}$	$7.4 \cdot 10^{-3}$	$2.5 \cdot 10^{-2}$	$4.7 \cdot 10^{-4}$	$7.4 \cdot 10^{-2}$	$1.2 \cdot 10^{-2}$	$2.5 \cdot 10^{-2}$	$6.1 \cdot 10^{-2}$
130	$5.9 \cdot 10^{-3}$	$7.4 \cdot 10^{-3}$	$2.3 \cdot 10^{-2}$	$4.7 \cdot 10^{-3}$	$7.5 \cdot 10^{-2}$	$1.1 \cdot 10^{-2}$	$2.4 \cdot 10^{-2}$	$7.1 \cdot 10^{-2}$
140	$5.4 \cdot 10^{-3}$	$6.3 \cdot 10^{-3}$	$2.3 \cdot 10^{-2}$	$5.3 \cdot 10^{-3}$	$7.6 \cdot 10^{-2}$	$1.3 \cdot 10^{-2}$	$2.4 \cdot 10^{-2}$	$7.6 \cdot 10^{-2}$
150	$4.7 \cdot 10^{-3}$	$6.7 \cdot 10^{-3}$	$2.5 \cdot 10^{-2}$	$5.2 \cdot 10^{-3}$	$7.8 \cdot 10^{-2}$	$1.5 \cdot 10^{-2}$	$2.2 \cdot 10^{-2}$	$7.7 \cdot 10^{-2}$
160	$4.7 \cdot 10^{-3}$	$6.5 \cdot 10^{-3}$	$3.0 \cdot 10^{-2}$	$5.8 \cdot 10^{-3}$	$8.2 \cdot 10^{-2}$	$1.7 \cdot 10^{-2}$	$2.1 \cdot 10^{-2}$	$7.8 \cdot 10^{-2}$
170	$5.0 \cdot 10^{-3}$	$4.7 \cdot 10^{-3}$	$4.1 \cdot 10^{-2}$	$6.2 \cdot 10^{-3}$	$8.4 \cdot 10^{-2}$	$1.7 \cdot 10^{-2}$	$2.2 \cdot 10^{-2}$	$7.6 \cdot 10^{-2}$
180	$5.5 \cdot 10^{-3}$	$4.0 \cdot 10^{-3}$	$4.9 \cdot 10^{-2}$	$6.4 \cdot 10^{-3}$	$8.9 \cdot 10^{-2}$	$1.7 \cdot 10^{-2}$	$2.3 \cdot 10^{-2}$	$7.4 \cdot 10^{-2}$
190	$6.4 \cdot 10^{-3}$	$3.2 \cdot 10^{-3}$	$5.6 \cdot 10^{-2}$	$6.5 \cdot 10^{-3}$	$8.8 \cdot 10^{-2}$	$2.0 \cdot 10^{-2}$	$2.4 \cdot 10^{-2}$	$7.2 \cdot 10^{-2}$
200	$7.1 \cdot 10^{-3}$	$2.6 \cdot 10^{-2}$	$5.8 \cdot 10^{-2}$	$6.8 \cdot 10^{-3}$	$9.4 \cdot 10^{-2}$	$2.3 \cdot 10^{-2}$	$2.6 \cdot 10^{-2}$	$6.5 \cdot 10^{-2}$

2. Supplementary Figures.

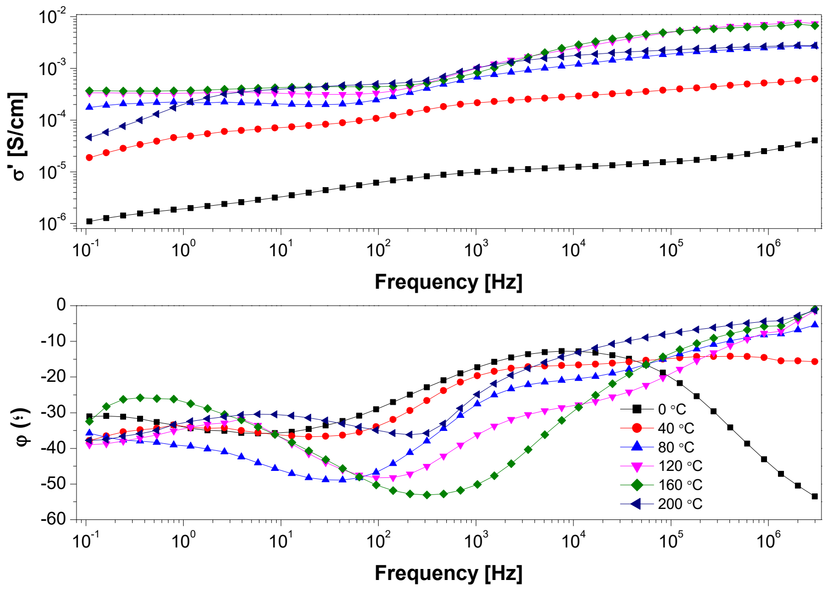

Figure S1. Bode diagram for phosphoric acid doped PBI@BMIM–Cl composite membrane (containing 5 wt. % of BMIM–Cl) under anhydrous conditions. In the top graphical representation σ′ is plotted against the frequency, whereas in the bottom, the out of phase angle ϕ is plotted against the frequency.

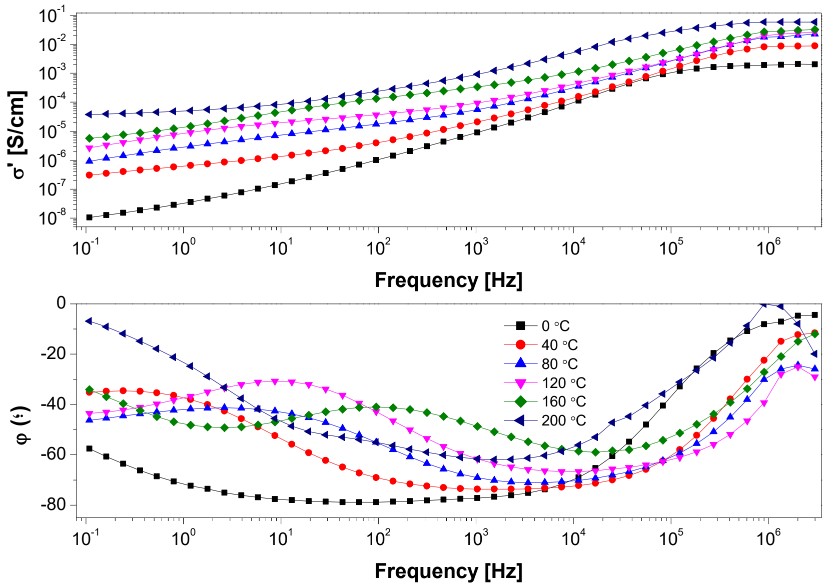

Figure S2. Bode diagram for phosphoric acid doped PBI@BMIM–Br composite membrane (containing 5 wt. % of BMIM–Br) under anhydrous conditions. In the top graphical representation σ′ is plotted against the frequency, whereas in the bottom, the out of phase angle ϕ is plotted against the frequency.

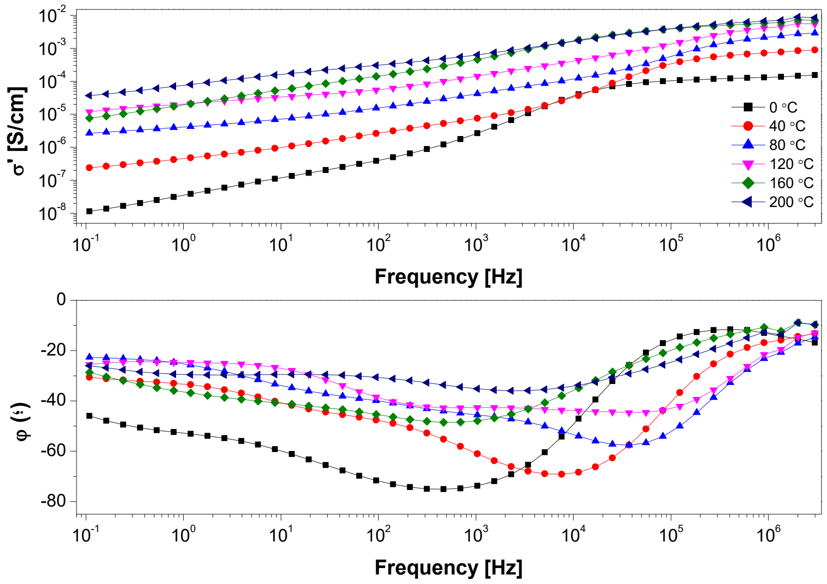

Figure S3. Bode diagram for phosphoric acid doped PBI@BMIM–I composite membrane (containing 5 wt. % of BMIM–I) under anhydrous conditions. In the top graphical representation σ′ is plotted against the frequency, whereas in the bottom, the out of phase angle ϕ is plotted against the frequency.

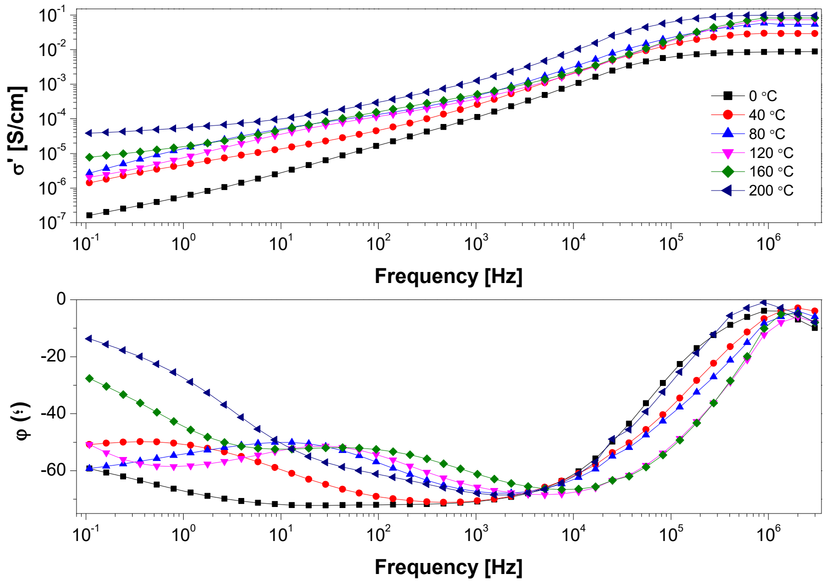

Figure S4. Bode diagram for phosphoric acid doped PBI@BMIM–BF_4 composite membrane (containing 5 wt. % of BMIM–BF_4) under anhydrous conditions. In the top graphical representation σ′ is plotted against the frequency, whereas in the bottom, the out of phase angle ϕ is plotted against the frequency.

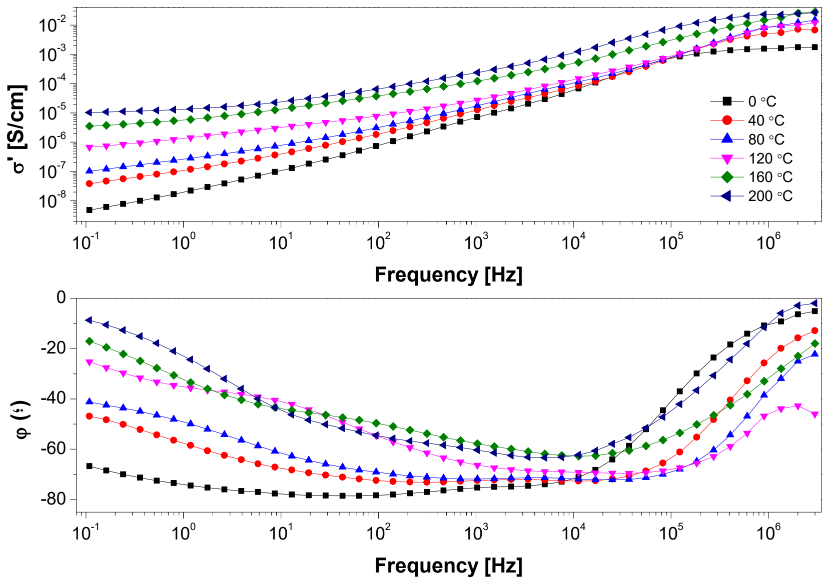

Figure S5. Bode diagram for phosphoric acid doped PBI@BMIM–PF_6 composite membrane (containing 5 wt. % of BMIM–PF_6) under anhydrous conditions. In the top graphical representation σ′ is plotted against the frequency, whereas in the bottom, the out of phase angle ϕ is plotted against the frequency.

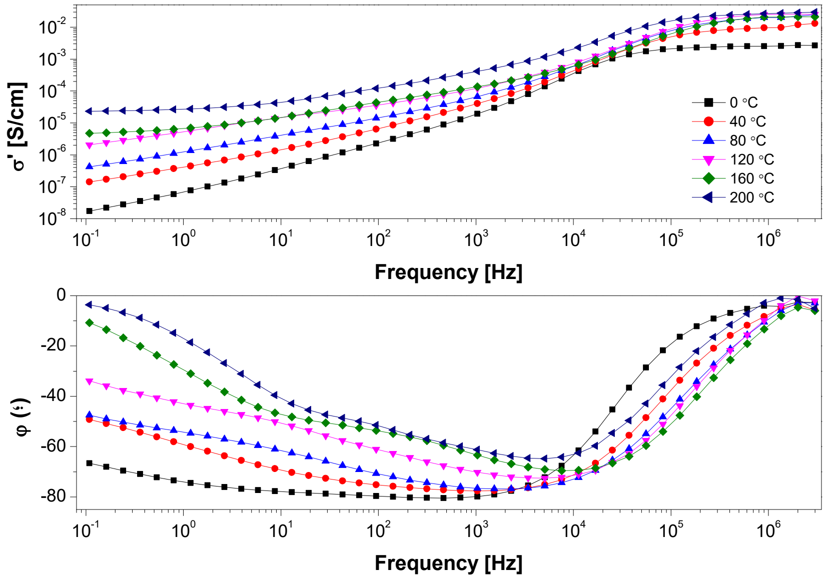

Figure S6. Bode diagram for phosphoric acid doped PBI@BMIM–NCS composite membrane (containing 5 wt. % of BMIM–NCS) under anhydrous conditions. In the top graphical representation σ′ is plotted against the frequency, whereas in the bottom, the out of phase angle ϕ is plotted against the frequency.
